# A rare case of non-functional Paraganglioma of urinary bladder with No LUTS

**DOI:** 10.1016/j.eucr.2025.102976

**Published:** 2025-02-03

**Authors:** Mohammad Poury, Mohsen Ayati, Seyed Ali Momeni, Laleh Sharifi

**Affiliations:** Uro-Oncology Research Center, Tehran University of Medical Sciences, Tehran, Iran

**Keywords:** Paraganglioma, Bladder, Lower urinary tract symptoms

## Abstract

Paraganglioma originates from chromaffin cells of the sympathetic nervous system. Paraganglioma of urinary bladder are very rare tumors that arise from the ganglion cells and usually mistakenly have been diagnosed and treated because of its rarity and diverse symptoms. Here, we present our patient with incidental finding of non-functional paraganglioma of urinary bladder who showed no lower urinary tract symptoms (LUTS). Timely diagnosis, treatment, and postoperative reviews are in effect for management of these patients.

## 1 Introduction

Paragangliomas are catecholamine-producing neoplasms arising from chromaffin cells of the sympathetic nervous system that are frequently diagnosed in an intradiaphragmatic position. Paraganglioma of bladder was described for the first time by Zimmerman et al., in 1953 as pheochromocytoma of the urinary bladder in an old woman.^1^ The tumor originates from the ganglion cells in the bladder wall, frequently on the trigone, with an average tumor size of 3.9 cm. Paraganglioma of urinary bladder is an infrequent tumor that represents only 0.06 percent of all urinary tumors and less than one-tenth of paragangliomas are found in the urinary bladder.^2^^,^^3^

According to their scarcity and the varying symptoms, paraganglioma of urinary bladder is usually mistakenly diagnosed and treated. Here, we present our patient with a non-functional paraganglioma of urinary bladder.

## 2 Case presentation

**Fig. 1:**
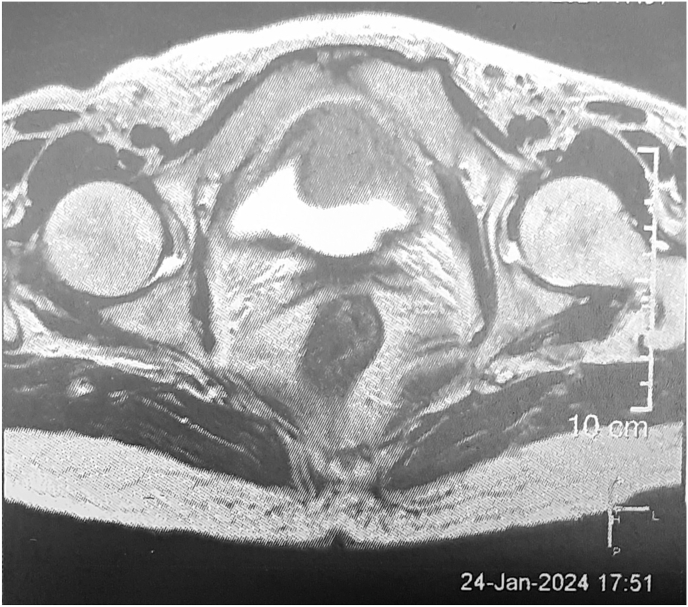
Magnetic resonance imaging of bladder mass Bladder mass was detected on the anterior wall of the urinary bladder with obvious enhancement and hyperintensity on T2-weighted imaging.

Here, we are presenting our patient who was a 72-year-old woman with an incidental finding of a heterogeneous mass on the floor of urinary bladder in an MRI study that was done for her hip joint problem. She had a history of lower limb pain and hypertension which was under control by regular use of Losartan. For precise study pelvic and abdomen MRI without and with IV gadolinium was carried out. Results showed 40∗28 lobulated intermediate signal intensity mass in T2w and low signal in T1w from the anterior wall of the urinary bladder with extension to the proximal part of the urethra. No sign of lymphadenopathy in the pelvic cavity or inguinal region was detected. After gadolinium injection, a marked enhancement without invasion out of the bladder was observed (Fig. 1). Chest CT scan showed no mass.

Transurethral resection of bladder tumor (TURBT) was done and microscopic evaluation of the specimen showed cells with pleomorphic and large granular nuclei and nucleoli without prominent mitotic figures and large eosinophilic granular cytoplasms in the vascular stroma. Some mucin-producing glands within the tumor were identified. In IHC test, Synaptophysin, chromogranin, vimentin and GATA3 were positive and CK (AE1/AE3), CK7, CK20, CAM5.2, EMA, S100 protein and CD68 were reported negative. Ki67 stain showed a very low proliferative index. Pathology determined Paraganglioma in the background of cystitis glandularis. Laboratory studies showed a normal range of urine and plasma catecholamines.

**Fig. 2:**
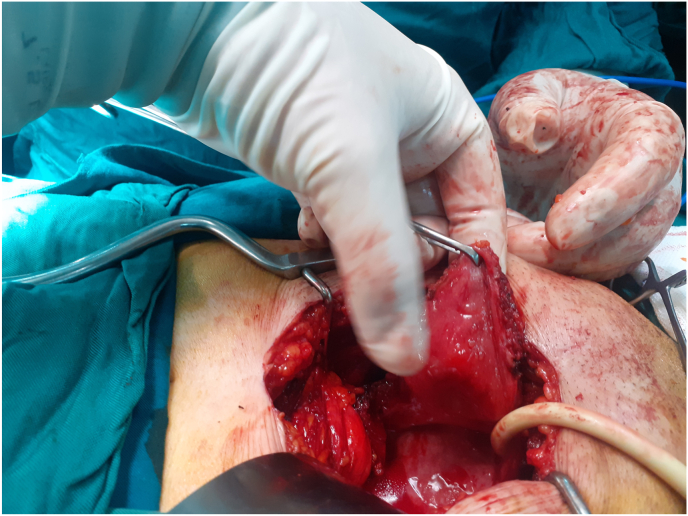
Operation field Paraganglioma mass was resected trough partial cystectomy.

**Fig. 3:**
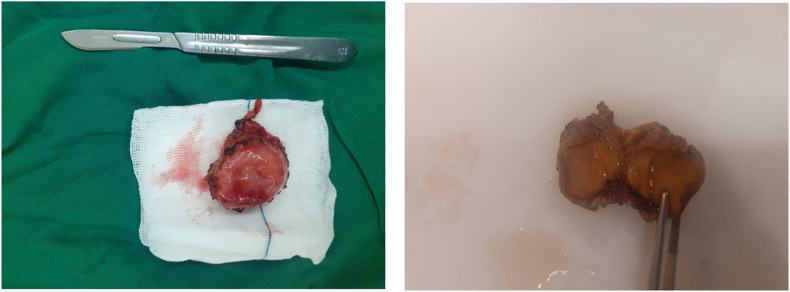
Resected Paraganglioma tumor.

Bladder mass was resected through partial cystectomy (Fig. 2, Fig. 3) and the after 2 days the patient was discharged in good condition. Six months later she was visited with no medical problem and advised to follow regularly.

## 3 Discussion

Development of paragangliomas as catecholamine-producing neuroendocrine tumors in urinary bladder is very unusual. It has been reported that young individuals are more prone to bladder paraganglioma. The tumors arise commonly in the trigon of the bladder with a mean size of 4 cm, variable depth and nature. Most patients usually LUTS triggered by urination or bladder overdistension, but in this case report we introduce an old woman with a paraganglioma tumor of anterior wall of bladder with no LUTS.

Currently there is no standard guideline for surgical management of bladder paraganglioma. Treatment varies based on the size of tumor, its location, and invasiveness as well as its functionality. In addition to lifelong follow-up, patients with bladder paraganglioma are typically treated by radical/partial cystectomy or transurethral resection.^4^ Mainly transurethral resection of bladder tumors is applicable for those lesser than 3 cm and can considerably decrease the hospital days after the surgery. Our patient underwent partial cystectomy according to the size of 4 cm.

Augmented secretion of endogenous catecholamines manifests symptoms of functional paragangliomas such as hypertension, headache, dyspnea, angina, and hematuria.^3^ According to the problematic diagnosis, imaging and blood tests are essential before surgery. Elevated levels of catecholamines including norepinephrine, adrenaline, and dopamine in blood or 24-h urine are important diagnostic tests to rule out the functional Paraganglioma of bladder.

Because of the rarity and the different symptoms, sixty percent of bladder paraganglioma masses were misdiagnosed before the pathologic study^5^ especially, non-functional tumors which do not show any clinical signs leading to misdiagnosis and challenges in treatment.^4^

Despite the lack of symptoms, fortunately an incidental finding of a bladder mass led to the diagnosis of paraganglioma in our patient. Our patient had a non-functional paraganglioma mass due to the normal levels of catecholamines before and after the tumor resection; also she still had blood pressure problems after removal of the tumor as she had to continue to use valsartan.

Timely diagnosis and treatment are in effect to management of the paitents with paraganglioma of bladder; Moreover, postoperative reviews are necessary to evaluate the efficiency of the treatment and tumor recurrence.

## CRediT authorship contribution statement

**Mohammad Poury:** Conceptualization, Investigation. **Mohsen Ayati:** Conceptualization, Supervision. **Seyed Ali Momeni:** Investigation, Writing – review & editing. **Laleh Sharifi:** Conceptualization, Supervision, Writing – original draft.

## Funding

No funding was received.

## Conflict of interest

The authors declare no conflict of interest.

## References

[1] Zimmerman I.J., Biron R.E., Macmahon H.E. (1953). Pheochromocytoma of the urinary bladder. N Engl J Med.

[2] Tiwari S.B., Ghimire B., Gautam K., Paudel R., Sharma N., Shrivastav S. (2021). Extra-adrenal paraganglioma of a urinary bladder in an adolescent male: a rare case report. Int J Surg Case Rep.

[3] Zhao Y., Zhang Z., Wang S. (2024). A retrospective study of paraganglioma of the urinary bladder and literature review. Front Surg.

[4] Hajji F., Benazzouz A., Hammoune N., Azami M.A., Ghoundale O. (2021 Oct 16). Functional bladder paraganglioma as an incidental finding during infertility workup. Cureus.

[5] Iwamoto G., Kawahara T., Tanabe M. (2017). Paraganglioma in the bladder: a case report. J Med Case Rep.

